# In Situ Microwave Ablation With Intralesional Resection and Subsequent Mechanical Reinforcement for Juxtaarticular Osteosarcoma Achieves Satisfactory Functional Outcomes: A Three-Year Kinematic Analysis

**DOI:** 10.5435/JAAOSGlobal-D-24-00404

**Published:** 2025-09-17

**Authors:** Wenhan Huang, Yuan Yan, Chongquan Huang, Jinpeng Lin, Yu Zhang

**Affiliations:** From the Department of Orthopaedics Oncology, Guangdong Provincial People's Hospital (Guangdong Academy of Medical Sciences), Southern Medical University (Dr. W. Huang, Dr. Yan, C. Huang, Dr. Lin, and Dr. Zhang); the School of Medicine, South China University of Technology (Dr. W. Huang, Dr. Yan, C. Huang, Dr. Lin, and Dr. Zhang); and the School of Materials Science and Engineering (National Engineering Research Center for Tissue Restoration and Reconstruction), South China University of Technology, Guangzhou, Guangdong, China (Dr. W. Huang, Dr. Y. Yan, Dr. C. Huang, and Dr. Lin).

## Abstract

**Background:**

Joint-preserving surgery of a patient's native knee joint for juxtaarticular osteosarcoma may enhance function but poses resection and reconstruction challenges.

**Methods:**

We included 15 patients with nonmetastatic distal femoral osteosarcoma who underwent in situ microwave ablation with intralesional resection and subsequent mechanical reinforcement. Knee function was assessed at 1 and 3 years postoperatively using Musculoskeletal Tumor Society scores, six degrees of freedom kinematic analysis. The 1-year and 3-year construct and overall survival rates were recorded. A control group of 20 healthy individuals was used for comparison.

**Result:**

At the 3-year follow-up, two patients had died. The final Musculoskeletal Tumor Society score was 29.0 ± 1.2 (range, 26 to 30). No major complication was recorded. At 1 year, notable gait differences were observed compared with healthy control subjects, including reduced knee flexion at 12%, 52%, 62%, 75%, and 85% of the gait cycle; increased adduction at 12%, 52%, 62%, and 75%; and increased external rotation at 52%, 62%, 75%, and 85%. The range of motion (ROM) in flexion/extension, internal/external rotation, proximal/distal translation, and medial/lateral translation (*P* < 0.05) were significantly reduced. At 3 years, most kinematic differences had diminished and ROM differences had largely resolved, with only an increase in internal/external and abduction-adduction ROM.

**Conclusion:**

The surgical procedure of in situ microwave ablation with intralesional resection and subsequent mechanical reinforcement shows restricted mobility at 1 year postoperatively, but knee kinematic performance is nearly indistinguishable from that of healthy individuals at the 3-year follow-up. With adequate resection and adjuvant treatment insured, mechanical reinforcement reconstruction effectively preserves knee function.

Osteogenic sarcoma or osteosarcoma predominantly originates in the metaphysis of long bones of adolescents and young adults, with approximately 43% in the distal femur.^1,2^ The introduction of chemotherapy significantly improved the prognosis for patients with localized osteosarcoma, elevating long-term survival rates from below 20% to between 65% and 70% because of multiagent chemotherapy regimens.^1,2^ Nowadays, limb salvage surgeries combined with neoadjuvant chemotherapy is an option for many patients. However, there is no universally accepted “best” method for reconstructing bone following the resection of an osteosarcoma tumor. The choice of optimal reconstruction technique depends on various factors including the tumor's location and size, the extent of functional epiphysis that can be preserved, the anticipated remaining growth, and the expertise available at the local center.^3-10^

Generally, endoprosthetic reconstruction offers a good chance for local control. Yet, endoprostheses come with their own set of complications postreconstruction. Reported long-term survival rates for prostheses range between 58% and 79.3%, often necessitating revisions, multiple surgeries, or additional treatments.^3-7^ For growing children, expandable prostheses are considered a promising option for managing limb length equality after epiphyseal resection. However, frequent revision surgeries (averaging 2.1-2.6 per patient), high complication rates (bone loss, infection, aseptic loosening, etc), and residual limb length discrepancies of 15 to 30 mm in over 60% of pediatric patients remain substantial challenges that cannot be overlooked.^8-10^

To achieve a durable reconstruction that ensures continued knee mobility, it is crucial to preserve the structural integrity of the knee joint and surrounding bone. Researchers are exploring various biological reconstruction methods to address this challenge effectively. These techniques include vascularized fibula autografts alone or in combination with an allograft,^11,12^ Masquelet bone-grafting technique,^13^ distraction osteogenesis reconstruction,^14^ and inactivated tumor-bearing bone transplantation with liquid nitrogen,^15^ autoclaving, pasteurization,^16^ adjuvant cyoablation,^17^ or adjuvant microwave ablation.^18^ However, auto/allografts offer bone stock that may benefit patients but necessitate extended periods of limited weight bearing and carry a known complication profile, including infection, nonunion, fracture, and the need revision surgery.^11-18^

The knee is one of the most complex joints in the human body, where the seemingly simple motions of extension and flexion are accompanied by a series of intricate locomotor patterns across different planes. Surgery inevitably involves damage to or removal of some healthy tissue, which can result in muscle weakness, contracture, pain, and other related symptoms. Although several studies have examined the kinematic and kinetic patterns in patients treated with wide resection and endoprosthetic reconstruction, detailed evaluations of biological reconstructions remain limited. Biological reconstruction is often purported to provide better functional outcomes, but few studies have meticulously tracked and reported on the long-term postoperative functional status of the knee joint.

In our study, we evaluated the effectiveness and safety of in situ microwave ablation with intralesional resection and subsequent mechanical reinforcement for osteosarcoma, while also recording postoperative six-degree-of-freedom (6DOF) knee kinematics. This technique allows for the preservation of native bone and periarticular joint structures. The outcomes were dynamically tracked at 1 and 3 years postoperatively, including Musculoskeletal Tumor Society functional score (MSTS), knee kinematic parameters during the gait cycle, and construct and overall survival outcomes. In addition, we recruited 20 healthy individuals as a control group to compare the kinematic parameters.

## Methods

### Patients

We prospectively included 15 patients with nonmetastatic juxtaarticular osteosarcoma in the distal femur (Supplementary 1, http://links.lww.com/JG9/A433) and retrospectively analyzed the outcomes. Twenty matched healthy individuals were recruited whose findings were compared with the patients' kinematic data, each individual has no history of any disorders or discomfort of lower extremities. The Ethical Committee of *** approved all the patient's written informed consent. The same surgeon performed all the surgeries. All patients received the standard neoadjuvant chemotherapy. The inclusion was as follows: (1) The lesions can be completely ablated and inactivated within the tumor segment (CT and MRI assessed), which requires a multipoint, fully covered ablation plan; (2) there was an available distance of at least 20 mm from the tumor edge to the articular surface, a criterion established to protect the articular cartilage from heat-related damage; and (3) no notable neurovascular involvement, pathologic fracture, and no distant metastasis were found.

### Surgical Procedure

**Figure 1:**
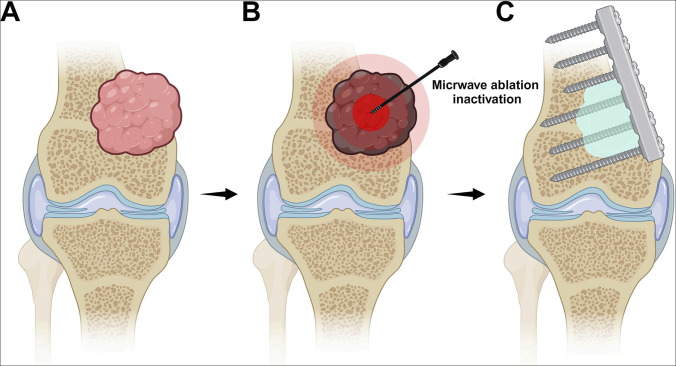
Images depicting the surgical illustration in this study. **A**, Distal femoral osteosarcoma. (**B**) Ablation-induced thermal necrosis of the entire tumor region. Black tumor indicates that the tumor has been inactivated. (**C**) Tumor removal by curettage or resection, followed by bone cement filling and internal fixation.

**Figure 2:**
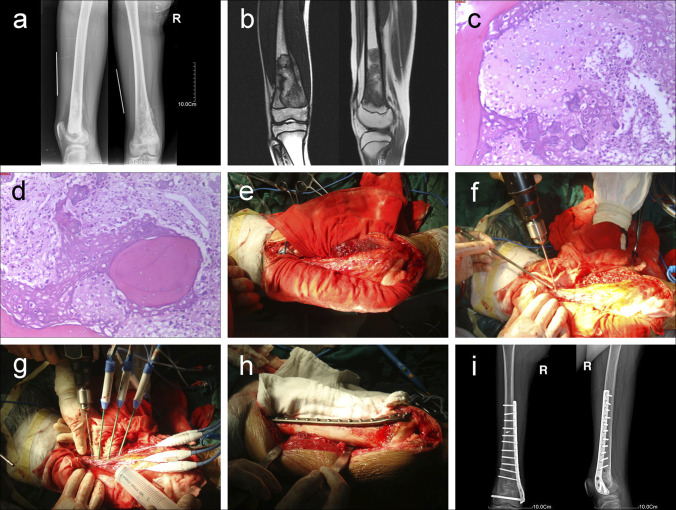
**A**–**I**, (**A**) AP and lateral radiographs and (**B**) MRI showing an osteosarcoma of the distal femur. **C** and **D**, Pathologic examination results of preoperative biopsy (**E**) separating the tumor-bearing segment from surrounding normal tissues and placing wet gauze between the tumor bone and surrounding normal tissues. **F** and **G**, Setting up the antenna array and performing en bloc MW ablation of the tumor segment, (**H**) the bone defect was filled with mixture of bone cement and bone graft, and prophylactic internal fixation was performed to strengthen the devitalized bone. **I**, Postoperative AP and lateral radiographs.

The surgical procedures involved in situ microwave ablation with intralesional resection and subsequent mechanical reinforcement, as detailed by Fan et al^19^ and Liu et al.^18,20^ The surgical illustration is shown in Figure 1, and a typical surgical case is presented in Figure 2. Before surgery, all patients underwent CT and MRI scans to delineate tumor margins. The most important was to identify the extent of the tumor and dissect the tumor-bearing bone from surrounding normal tissues with a safe margin (at least 20 mm width) for good exposure. In this cohort, all patients underwent a medial approach incision, and the vastus medialis muscle and partial or total vastus intermedius muscle were removed. Then, an ablation area is constructed to completely inactivate the tumor in situ by Microwave Ablation System (VISON Medical). Regions with intermediate signal intensity adjacent to the tumor edge were deemed part of the tumor and thus included in the ablation area, and the ablation needles were arranged to form a matrix area. Through intraoperative fluoroscopy, the ablation needle tips are positioned 1.5 cm apart (microwave ablation array with antennas and interval determined by the radiation range of ablation system used), ensuring that the ablation zones fully cover the tumor area, with temperatures in the ablation zone reaching at least 70°C. The area of ablation was evaluated strictly by radiologists and surgeons: Area of tumor response, progression, and suspicious invasion (intermediate-high signal and edema range in T2) were assessed. As soon as the ablation began, ice saline was squirted by the injector on the wet gauze to make sure the temperature of normal tissues was below 40°C. Wide margins cutoff of at least 2 cm width or more over the normal tissue around the tumor were obtained. Intraoperative frozen section examination was performed on the peripheral margins and confirmed that it was negative for viable tumor. When the tumor is more than 1 cm away from the articular cartilage, ablation is used to treat the tumor edge, followed by bone resection.^18,20^ Finally, a mixture of bone cement and prophylactic internal fixation was used to fill the defect and enhance mechanical support. Patients began a rehabilitation program once the wound had healed and wore a protective brace after surgery to prevent mechanical complications.

### Kinematic Data Collection

**Figure 3:**
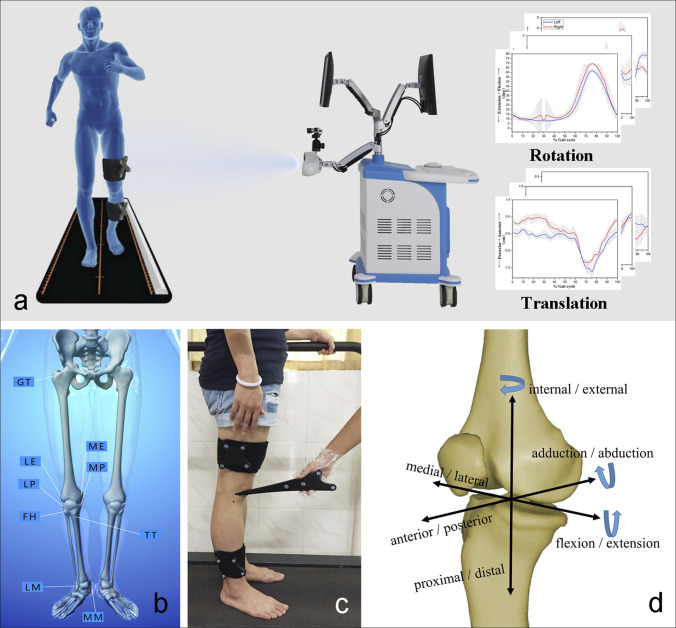
**A**–**D**, Images demonstrating (**A**) the portable optical tracking system and process of kinematics analysis. **B** and **C**, The identification of the lower limb anatomic landmarks was performed using a handheld probe before the kinematic data was captured. **D**, Definition of local femoral and tibial coordinate systems.

Kinematic parameters were collected at the motion analysis laboratory (Figure 3). Patients' gait kinematics were routinely collected at 1 year (n = 15) and 3 years (n = 12; three patients died of the disease) after surgery to evaluate the limb function and implement patient education. The kinematic assessment was performed through a novel optical motion analysis system (Opti-Knee, Innomotion). Three-dimensional (3D) spatial orientation was identified by an integrated 2-head stereo-infrared camera (NDI Polaris Spectra; Northern Digital) with an accuracy of 0.3-mm root mean square.^21^ Previous research has reported the feasibility and accuracy of this technique.^22,23^

Before data collection, patients or healthy subjects stood in a static reference pose with feet parallel and shoulder-width apart. Two rigid plates, each composed of four infrared retroreflective markers (OK_Marquer; Innomotion), were attached to the lateral aspect of the thigh and lower leg respectively with a band. Afterward, a digitalizing probe embedded with four infrared retroreflective markers was used to identify lower extremity landmarks (ie, greater trochanter, lateral femoral epicondyle, medial femoral epicondyle, lateral tibial plateau, medial tibial plateau, tibial tuberosity, fibular head, medial malleolus, lateral malleolus). Based on this static standing trial, anatomic frames on the femur and tibia were defined according to the bone landmarks. Patients were instructed to walk on a horizontal treadmill at a preferred speed for 3 to 5 minutes to ensure the gait pattern was analogous to normal over-the-ground gait. Then the gait cycles were subsequently captured for 15 seconds at a frequency of 60 Hz and approximately 20 gait cycles were collected. The 3D translation was quantified as the relative displacement between the origins of the tibial and the femoral coordinates. Similarly, the angular rotations were determined by the femoral coordinate system with respect to the tibial coordinate system using Euler angles in the following sequence: mediolateral axis, anterior-posterior axis, and proximodistal axis.^23^ Both knees underwent a similar procedure for motion capture on the bidirectional treadmill. A customized MATLAB was used to analyze the kinematics data of each cycle and interpolate each gait cycle to 100 points. 6DOF knee kinematics were analyzed, including adduction/abduction, internal/external tibial rotation, flexion/extension, anterior/posterior translation, proximal/distal translation, and medial/lateral tibial translation. Gait cycles were divided into two phases: (1) the stance phase (∼62% of the gait); and (2) the swing phase (63% to 100% of the gait). Motion patterns and the range of motion (ROM) in 6DOF were analyzed by calculating the difference between the maximum and minimum values. The key events were collected, including opposite toe off (loading response, 12% of gait), heel rise (middle stance, 31% of gait), opposite initial contact (terminal stance phase, 52% of gait), toe off (preswing phase, 62% of gait), feet adjacent (initial swing phase, 75% of gait) and tibial vertical (mid-swing phase, 87% of gait). The selection of these key points referenced fundamental concept of Rancho Los Amigos gait phase classification method,^24^ and explanations and clarifications are provided in the Supplementary 2, http://links.lww.com/JG9/A434.

### Statistical Analysis

All values were expressed as mean ± SD, and all error bars represented the SD of the mean. The kinematic characteristics of patients and healthy participants were compared using one-way analysis of variance for parametric variables, and the Mann–Whitney test was used for nonparametric variables. Nonnormalized data were fitted by multiple linear regression models in an attempt to establish the associations among the side of the affected limb, age, sex, body mass index, and tumor volume for each key kinematic parameter of the gait cycle. All statistical tests were two-sided. A probability less than 0.05 was considered statistically significant. Statistical analyses were performed using SPSS Version 19.0 (SPSS).

## Results

The flowchart of inclusion and exclusion is illustrated in Supplementary 1, http://links.lww.com/JG9/A433. The study recruited 15 patients and 20 healthy subjects. None of the 15 patients experienced local recurrence; however, three patients developed lung metastases and subsequently passed away, while the remaining 12 patients had no recurrence or metastasis. The characteristics of patients are shown in Table 1. The patient group had a mean age of 22.9 ± 13.3 years (27.2 ± 14.0 at 3-year follow-up), a mean body mass index of 19.6 ± 1.9 kg/m^2^ (19.3 ± 1.9 at 3-year follow-up), and a sex distribution of six female to nine male patients (five female to seven male patients at 3-year follow-up). No significant differences were observed in demographics between the two groups (Supplementary 3, http://links.lww.com/JG9/A435). The cohort showed an MSTS score of 29.0 ± 1.2, with a mean tumor volume of 137.8 ± 91.9 cc. No severe complications were reported in this cohort, including notable wound healing complications requiring secondary surgery, deep infections, implant failure, or long-term fragility fractures. Bone scans revealed partial epiphyseal osteonecrosis in two patients, but both remained asymptomatic.

**Table 1:** Characteristics of Patients

Patient	Age	Sex	BMI (kg/m^2^)	Side	MSTS	Tumor Volume (cc)
1	17 (19)	Male	21.78 (20.82)	Right	30	260.79
2	19 (21)	Male	21.72 (20.32)	Left	30	341.42
3	15 (17)	Female	20.45 (19.22)	Right	28	22.22
4	23 (25)	Male	19.72 (19.38)	Left	30	58.02
5	25 (27)	Female	19.33 (19.71)	Left	30	12.77
6^a^	10	Male	16.07	Left	None	130.58
7	18 (20)	Male	18.65 (19.13)	Left	29	206.20
8	18 (20)	Male	21.97 (21.30)	Left	29	92.52
9	14 (16)	Male	16.63 (16.44)	Right	29	236.51
10^a^	19	Male	18.87	Right	None	56.71
11	22 (24)	Female	18.96 (18.59)	Right	29	102.46
12	47 (49)	Female	19.15 (18.31)	Right	28	143.58
13^a^	13	Female	19.77	Right	None	116.92
14	60 (62)	Male	22.51 (22.51)	Left	26	168.71
15	24 (26)	Female	17.85 (16.02)	Left	30	116.92

MSTS = Musculoskeletal Tumor Society functional score

a These patients were died within 3 years.

The MSTS was evaluated at 3 years postsurgery.

**Figure 4:**
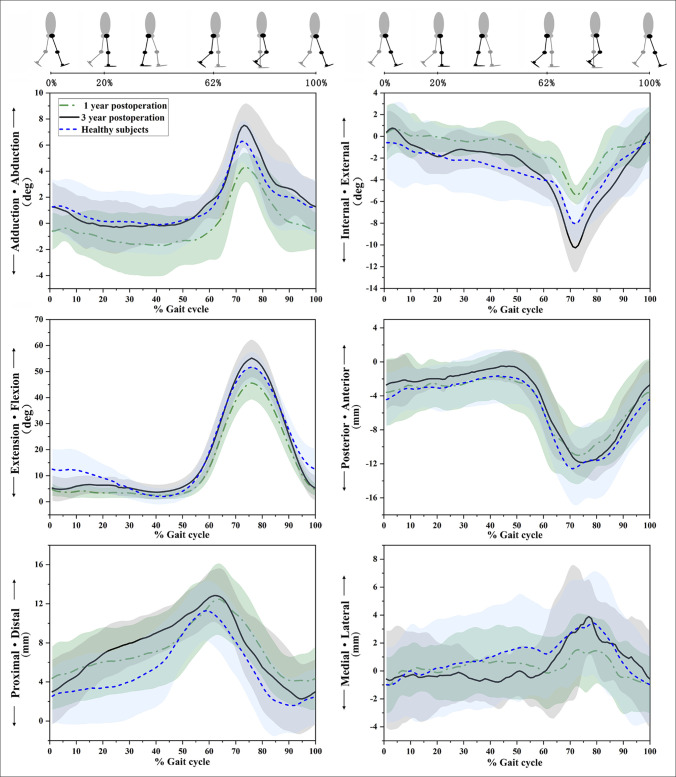
Images demonstrating three-dimensional knee joint rotations and translations during the gait cycle. The green dash-dotted line, black solid line, and blue dashed line represent 1 year postsurgery, 3 years postsurgery, and healthy subjects, respectively. Shaded areas indicate the standard deviation of healthy subjects.

The key points of 6DOF kinematic parameters in 1 year (n = 15) and 3 years (n = 12) postoperatively compared with healthy subjects were summarized in Table 2 and curves are illustrated in Figure 4. Interestingly, at 1 year postsurgery, notable differences were observed throughout the entire gait cycle compared with healthy subjects, including a reduction in knee flexion angle at 12%, 52%, 62%, 75%, and 85% of the gait cycle; increased adduction at 12%, 52%, 62%, and 75%; and increased external rotation at 52%, 62%, 75%, and 85%. No notable difference was observed in AP, proximal-distal, and medial-lateral translation.

**Table 2:** 6 DOF Kinematics of Patients and Healthy Subjects During Treadmill Gait

Factor or Variable	Gait Cycle				
	12%	52%	62%	75%	85%
Flexion(+)-extension(−), deg					
1 year postsurgery	4.2 ± 2.5^a^	3.8 ± 1.9	17.9 ± 4.5^a^	45.3 ± 6.2^a^	33.9 ± 5.7^a^
3 years postsurgery	6.3 ± 3.6^a^	6.8 ± 3.5	23.7 ± 7.0	54.6 ± 7.2	40.7 ± 4.7
Healthy subjects	11.7 ± 7.1	5.4 ± 3.3	24.6 ± 5.1	51.4 ± 5.8	39.9 ± 5.1
Adduction(−)-abduction(+), deg					
1 year postsurgery	−0.8 ± 1.6^a^	−1.3 ± 1.8^a^	−0.3 ± 2.5^a^	4.2 ± 1.1^a^	1.1 ± 2.3
3 years postsurgery	0.1 ± 1.9	0.2 ± 1.4	1.9 ± 1.6	7.1 ± 1.9^a^	3.0 ± 3.1
Healthy subjects	0.6 ± 2.4	0.3 ± 1.8	1.5 ± 1.7	5.8 ± 1.7	2.3 ± 2.6
Internal(−)-external(+), deg					
1 year postsurgery	0.1 ± 1.9	−1.1 ± 1.6^a^	−1.9 ± 1.5^a^	−4.7 ± 1.1^a^	−1.3 ± 2.1^a^
3 years postsurgery	−0.9 ± 1.3	−2.3 ± 2.0	−4.4 ± 1.3	−8.8 ± 2.3^a^	−4.9 ± 2.1
Healthy subjects	−1.5 ± 2.8	−3.5 ± 2.4	−4.1 ± 2.3	−7.0 ± 1.8	−3.3 ± 3.1
Anterior(−)-posterior(+), mm					
1 year postsurgery	−2.9 ± 3.1	−2.5 ± 3.5	−7.4 ± 4.8	−10.9 ± 3.1	−8.6 ± 3.4
3 years postsurgery	−2.3 ± 1.5	−1.1 ± 2.2	−6.3 ± 3.6	−11.8 ± 1.7	−10.0 ± 2.5
Healthy subjects	−3.2 ± 2.4	−2.2 ± 2.8	−7.5 ± 3.0	−11.9 ± 4.3	−10.7 ± 4.0
Proximal(+)-distal(−), mm					
1 year postsurgery	5.5 ± 3.4^a^	9.6 ± 3.8	12.3 ± 3.6	9.4 ± 3.1	5.2 ± 2.6
3 years postsurgery	5.1 ± 2.9	10.9 ± 2.4^a^	12.8 ± 2.7	7.4 ± 3.5	4.2 ± 4.5
Healthy subjects	3.0 ± 3.3	8.1 ± 3.3	11.2 ± 3.0	7.7 ± 3.4	3.1 ± 4.0
Medial(−)-lateral(+), mm					
1 year postsurgery	0.2 ± 1.8	0.5 ± 2.6	−0.1 ± 2.6	1.3 ± 2.7	0.8 ± 2.4
3 years postsurgery	−0.3 ± 3.1	−0.1 ± 1.9	0.3 ± 3.6	3.3 ± 3.0	2.0 ± 2.7
Healthy subjects	−0.2 ± 3.4	1.7 ± 3.8	1.3 ± 3.1	3.1 ± 3.5	2.1 ± 3.9

a A statistically significant difference compared with healthy subjects (*P* < 0.05).

At 3 years postoperatively, most kinematic differences essentially disappeared, with no statistically significant differences remaining except for the following: (1) Flexion angle at 12% of the gait cycle was still reduced by 5.4° (6.3 ± 3.6° in patients vs. 11.7 ± 7.1° in healthy subjects, *P* < 0.05); (2) abduction angle at 75% of the gait cycle was increased by 1.3° (7.1° ± 1.9° in patients vs. 5.8° ± 1.7° in healthy subjects, *P* < 0.05); (3) internal rotation at 75% of the gait cycle was increased by 1.8° (−8.8° ± 2.3° in patients vs. −7.0° ± 1.8° in healthy subjects, *P* < 0.05); and (4) proximal translation at 52% of the gait cycle was increased by 2.8 mm (10.9 ± 2.4 mm in patients vs. 8.1 ± 3.3 mm in healthy subjects, *P* < 0.05). All other kinematic parameters at 3 years postoperatively were not significantly different from those of healthy subjects.

**Figure 5:**
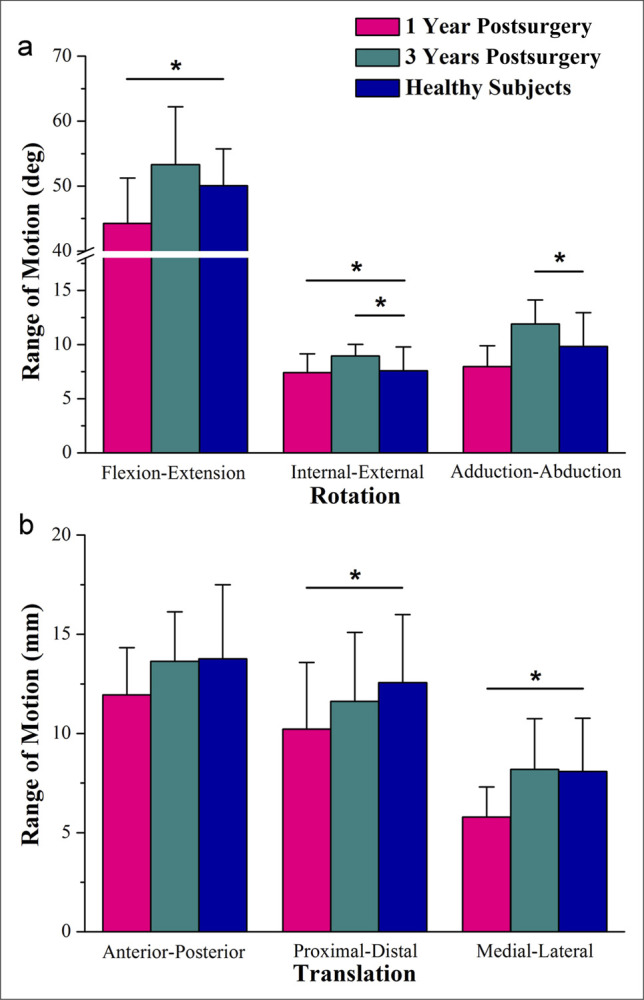
Graph showing range of motion (mean ± SD) of the kinematic parameters of the 1 year postsurgery, 3 years postsurgery, and healthy subjects during the treadmill kinematic test: (**A**) rotation and (**B**) translation. *Statistically significant difference (*P* < 0.05).

The ROM of the 6DOF kinematics is shown in Figure 5. At 1-year postsurgery, the ROM of flexion/extension angle (*P* = 0.005) and internal/external angle (*P* = 0.019) were significantly smaller than observed in healthy subjects. Similarly, proximal/distal translation (*P* = 0.035) and medial/lateral translation (*P* < 0.001) exhibited smaller significantly compared with healthy subjects. However, at 3 years postsurgery, the differences in ROM had disappeared, with only an increase in ROM observed in internal/external (*P* = 0.043) and adduction-abduction (*P* = 0.001).

Supplementary 4 and 5, http://links.lww.com/JG9/A436 and http://links.lww.com/JG9/A437 provided the correlations between kinematic features, ROM, and baseline data of patients in 1 year postsurgery and 3 years postsurgery, respectively.

## Discussion

To our knowledge, this is the first report of a series on 6DOF knee kinematic changes in oncological patients undergoing joint-sparing surgery with biological reconstruction. We demonstrated that patients with osteosarcoma who undergo joint-sparing surgery can achieve functional improvement and the kinematic performance of the gait cycle similar to healthy subjects. Although we observed “restricted mobility” and kinematic differences across various degrees of freedom in the 1-year postoperative follow-up, these differences diminished in medium- to long-term follow-up.

Previous studies have shown that different reconstruction methods all achieve good lower limb function, with the median/average MSTS score from 23.1 to 28 (23 to 25, vascularized autografts^12,16,25^; 23.1, pasteurized autografts^16^; 27.8, cryoablation^17^; 28, microwave ablation^18^). In our study, the MSTS score was 29.0 ± 1.2 (from 26 to 29), which was similar to previous studies. The satisfied MSTS scores can be attributed to the well-preserved intraarticular structure and the retained osseous bed, which allowed for the effective reattachment of soft tissues. In this study, our technique differs from the traditional resection approach by using adjuvant in situ ablation to achieve an effect similar to “radical resection.” In addition, compared with conventional biological reconstruction—such as allograft reconstruction or reimplantation of a devitalized autograft following complete tumor resection and ex vivo treatment (eg, microwave or cryotherapy)^11-18^—this technique offers distinct biomechanical and anatomical advantages. The preservation of native bone and periarticular joint structures, including the articular cartilage, plays a crucial role in achieving superior kinematic outcomes. It provided perfect size matching and collateral ligament preservation, and especially to the preserved articular cartilage surface, thereby enhancing postoperative knee function. This may explain why this cohort demonstrates better long-term function. In addition, our findings establish a standardized kinematic data set, providing a valuable reference for future comparisons with other resection techniques incorporating biological or mechanical reconstruction.

From a technical perspective, the complications of ablation for bone tumors are primarily associated with thermal injury, including superficial skin and flap burns or healing disturbances, bone necrosis, increased bone fragility and subsequent fracture, and neurovascular damage.^18-20,26^ In our cohort, these complications were not observed, potentially because of the following reasons. With adequate exposure—when the surgical field is fully exposed—it is easier to protect surrounding tissues using ice saline irrigation. Considering the femur is a major weight-bearing bone, prophylactic internal fixation and/or bone cement were always used. The presence of well-vascularized muscle coverage provided additional protection.

To quantitively evaluate limb function after limb salvage surgery, various optical navigation-based instruments have been used in previous works.^27-31^ These studies focused on the effects following prosthesis replacement and/or partial muscle resection. However, most studies focused on the type of endoprostheses and were heterogeneous regarding methodology, pathology, and site of lesion (tibia or femur).^27-31^ For example, Benedetti et al^27^ evaluated the limb function of patients after implantation of a modular hinged noncemented knee prosthesis. Although the functional outcome assessment score was similarly good for all patients, they found differences in gait biomechanics depending on which part of the quadriceps had been excised. Okita et al^30^ in a gait analysis demonstrated that patients tended to rely on the bilateral hip, ankle, and contralateral knee to generate additional power to increase walking speed, which is a critical capacity related to quality of life. Objective functional assessment does provide valuable insight into the in vivo limb function and helps improve limb function. Clinically, kinematic outcomes could provide information beyond imaging and physical examination and assist in developing personalized treatment and rehabilitation plans.

In our study, it is noteworthy that all patients were able to walk without any assistive devices at the 1-year follow-up. However, they showed a relatively “restricted” gait pattern with reduced ROM in most degrees of freedom, consistent with the key points from kinematics parameters. These kinematic alternations are believed to be multifactorial but anticipated. Joint conglutination caused by wearing a protective brace and disuse atrophy of muscle group around the knee, dystrophy suffering from postoperative chemotherapy, or psychological factors of the affected limb, and others may contribute to the unsatisfying kinematic performance at 1st year follow-up.^32,33^ Anyway, these factors are generally reversible and tend to improve gradually through rehabilitation exercises and long-term recovery. This explains why the kinematic differences observed in our study nearly disappeared by the 3rd year.

However, some kinematic differences between patients and matched healthy subjects were still detected in a 3-year follow-up. In the sagittal plane, 3-year kinematic outcomes revealed a markedly higher degree of knee extension during the load response phase (6.3 ± 3.6 vs. 11.7 ± 7.1; flexion (+), 12% of the gait cycle) compared with healthy subjects. Mechanically, full knee extension provides the benefit of knee stability. According to kinesiology, during non–weight-bearing knee extension, the femur rotates medially on the tibia, and the last degrees of spin lock the knee in extension. With quadriceps weakness, the patients may lean the body anteriorly over the quadriceps at the initial stance phase and the center of gravity falls in front of the knee.^32,34^ During non–weight-bearing knee extension, the femur rotates medially on the tibia, locking the knee in the final degrees of extension. With quadriceps weakness, a patient may lean forward at the initial stance phase, shifting the center of gravity in front of the knee, which forces the knee into extension.^32,34^ Another compensation involves the gluteus maximus and gastrocnemius stabilizing the femur, pulling the knee into extension at heel strike. This pattern of gait in the initial stance phase has been described by previous literature, in which the authors also stressed the importance of quadriceps.^27,28^ In addition, in the terminal stance phase (52% of the gait cycle), an increased tibial proximally translation was observed compared with the healthy subjects (10.9 ± 2.4 vs. 8.1 ± 3.3, *P* < 0.05). The terminal stance phase in the gait cycle is when the body's weight is nearly fully supported by a single leg, making it one of the phases where the load on the lower limb is at its maximum. Li et al^18^ reported a degenerative rate of 27% in patients who underwent MW-aided joint salvage resections for juxtaarticular osteosarcoma. It remains unclear whether the degenerative changes in the meniscus, potentially caused by heat damage, have affected its buffering capacity. Latent chondral degeneration deserves further investigation. Moreover, the larger ROM of adduction/abduction and internal/external rotation were observed at 3 years after surgery (*P* < 0.05). The increased rotational movements reflect the laxity in the patient's knee joint. Owing to the need to expose and protect the medial collateral ligament during surgery (especially thermal damage), it is not uncommon to detach and then reconstruct it.^35,36^ The partial excision of vastus medials may be responsible for this kinematic change as well.

We acknowledge the limitations of this study. First, the rarity of osteosarcoma and the approach to reconstruction surgical procedures limits the sample size. Second, the lack of kinetic analyses and electromyographic analyses may affect the comprehensive understanding of kinematic results. Third, treadmill gait may differ from gait on the ground, despite a previous study justifying the use of treadmill over-ground gait studies. Treadmill gait provided standardized walking speed and enhanced the comparability of results. Fourth, a 3-year follow-up was relatively short for the patients with malignant tumors. Additional studies should be performed to evaluate the relationship between the knee kinematics, mechanics of both grafts and types of internal fixation, and the walking gait over a longer follow-up period. Our study did not assess the tumor necrosis rate, and strictly speaking, there is no direct histologic evidence supporting whether the tumor region was completely inactivated, despite negative results from the biopsy of peripheral incisal margins.^18^ In addition, this study did not differentiate the distance of the surgical margin from the transepiphyseal resection, nor did it perform a subgroup analysis.

## Conclusion

This study indicates that the patients who underwent in situ microwave ablation with intralesional resection and subsequent mechanical reinforcement show restricted mobility at 1 year postoperatively, but knee kinematic performance is nearly indistinguishable from that of healthy individuals at the 3-year follow-up. With adequate resection and adjuvant treatment insured, mechanical reinforcement reconstruction effectively preserves knee function.

## Supplementary Material
