# Supplementary figures and images for: In Situ Microwave Ablation With Intralesional Resection and Subsequent Mechanical Reinforcement for Juxtaarticular Osteosarcoma Achieves Satisfactory Functional Outcomes: A Three-Year Kinematic Analysis
Source: J Am Acad Orthop Surg Glob Res Rev. 2025 Sep 17;9(9):e24.00404. doi: 10.5435/JAAOSGlobal-D-24-00404 (PMC12445414; doi:10.5435/JAAOSGlobal-D-24-00404)

**Supplementary 1** Flowchart of the inclusion and exclusion.


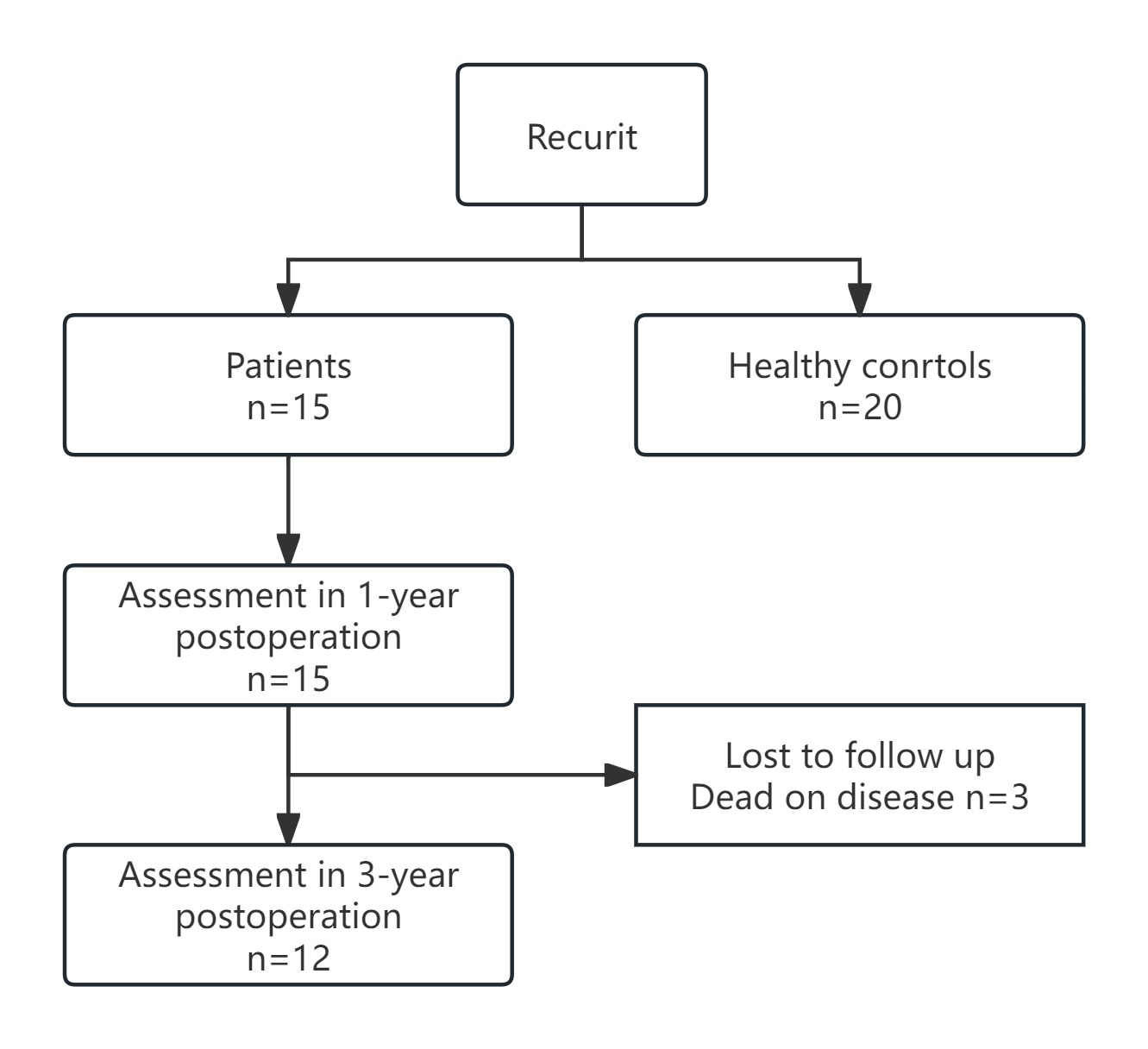

Supplement: Supplementary file 1 [file jagrr-9-e24.00404-s001.doc]
