# Supplementary material for: In Situ Microwave Ablation With Intralesional Resection and Subsequent Mechanical Reinforcement for Juxtaarticular Osteosarcoma Achieves Satisfactory Functional Outcomes: A Three-Year Kinematic Analysis
Source: J Am Acad Orthop Surg Glob Res Rev. 2025 Sep 17;9(9):e24.00404. doi: 10.5435/JAAOSGlobal-D-24-00404 (PMC12445414; doi:10.5435/JAAOSGlobal-D-24-00404)

**Supplementary 2** Key points referenced fundamental concept of Rancho Los Amigos gait phase classification method

Gait Analysis: Normal and Pathological Function" is a classic work in the field of gait analysis, co-authored by Jacqueline Perry and Judith M. Burnfield.This book adopts the Rancho Los Amigos (RLA) gait phase classification method, which divides the gait cycle into eight phases and categorizes them based on functional tasks. This classification method has been widely recognized by biomechanical researchers. Reciprocal Floor Contact Patterns: A gait cycle (GC) refers to the sequence in which one limb serves as a mobile support while the contralateral limb advances to a new support position. The roles of the limbs alternate, ensuring a smooth transfer of the body's center of gravity to the new supporting foot. The gait cycle consists of the Stance Phase, when the foot is on the ground, starting at Initial Contact (IC), and the Swing Phase, when the foot is in the air, beginning at Toe-Off (TOE-OFF). This method involves dividing the entire gait cycle into 1-100% time points for analysis. The focus of the analysis is on the support phase, as the swing phase belongs to the non-load-bearing stage. Time Distribution in Normal Gait: The stance phase constitutes approximately 60% of the gait cycle. The swing phase accounts for about 40%. Simply put, the following are currently commonly used: opposite toe off (loading response, 12 % of gait), heel rise (middle stance, 31 % of gait), opposite initial contact (terminal stance phase, 52 % of gait), toe off (pre-swing phase, 62 % of gait), feet adjacent (initial swing phase, 75 % of gait) and tibial vertical (mid-swing phase, 87 % of gait)


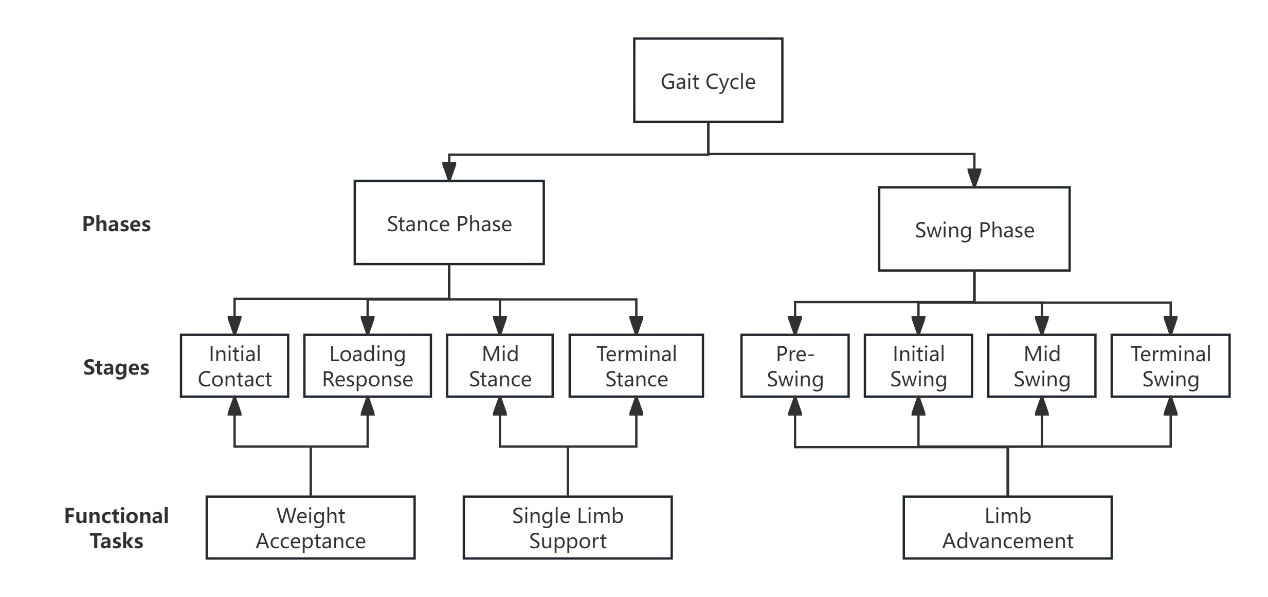


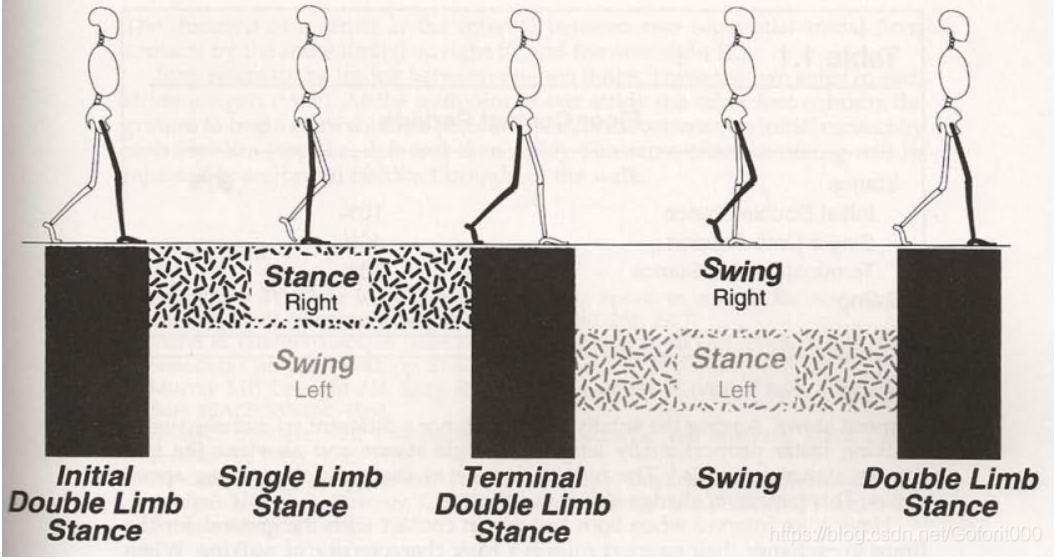

Supplement: Supplementary file 2 [file jagrr-9-e24.00404-s002.doc]
