# Supplementary material for: In Situ Microwave Ablation With Intralesional Resection and Subsequent Mechanical Reinforcement for Juxtaarticular Osteosarcoma Achieves Satisfactory Functional Outcomes: A Three-Year Kinematic Analysis
Source: J Am Acad Orthop Surg Glob Res Rev. 2025 Sep 17;9(9):e24.00404. doi: 10.5435/JAAOSGlobal-D-24-00404 (PMC12445414; doi:10.5435/JAAOSGlobal-D-24-00404)
Supplement: Supplementary file 3 [file jagrr-9-e24.00404-s003.doc]

**Supplementary 3** Demographics of patients and healthy subjects

|  | Patient group | healthy group |
| --- | --- | --- |
| age (years) | 22.9±13.3 (27.2±14.0)^a^ | 26.2±13.3 |
| BMI (kg/m^2^) | 19.6±1.9 (19.3±1.9) ^a^ | 19.7±3.5 |
| sex (female/male) | 6/9 (5/7) ^a^ | 8/12 |

^a^ Because three patients died between 1 to 3 years after surgery, the average age changed and recalculated as in the round bracket
