# Supplementary material for: In Situ Microwave Ablation With Intralesional Resection and Subsequent Mechanical Reinforcement for Juxtaarticular Osteosarcoma Achieves Satisfactory Functional Outcomes: A Three-Year Kinematic Analysis
Source: J Am Acad Orthop Surg Glob Res Rev. 2025 Sep 17;9(9):e24.00404. doi: 10.5435/JAAOSGlobal-D-24-00404 (PMC12445414; doi:10.5435/JAAOSGlobal-D-24-00404)
Supplement: Supplementary file 4 [file jagrr-9-e24.00404-s004.doc]

**Supplementary 4 Key point of ROM and 6DOF that characterize the one-year postoperative kinematic profiles, and their correlations with affected side, sex, age, BMI and Tumor Volume. Beta coefficients are calculated relative to the directions of the kinematic curves, (see Figure 2). Bolded p-values indicate statistical significance with the Bonferroni threshold.**

| Parameter | R^2^ | Side | | Sex | | Age(yrs) | | BMI(kg/m^2^) | | Tumor Volume (CC) | | |
| --- | --- | --- | --- | --- | --- | --- | --- | --- | --- | --- | --- | --- |
|  |  | *β* | *p* | *β* | *p* | *β* | *p* | *β* | *p* | *β* | *p* | |
| **ROM** | | | | | | | | | | | | |
| AB/AD | 0.308 | 1.949 | **0.040** | -0.775 | 0.386 | -0.289 | 0.255 | -0.344 | 0.165 | -0.097 | 0.742 | |
| IR/ER | 0.123 | 0.864 | 0.436 | 0.762 | 0.499 | -0.351 | 0.217 | 0.206 | 0.476 | 0.176 | 0.593 | |
| F/E | 0.073 | 3.773 | 0.362 | -2.066 | 0.620 | 0.432 | 0.133 | 0.063 | 0.833 | 0.122 | 0.719 | |
| A/P | 0.041 | 0.614 | 0.664 | 0.539 | 0.708 | -0.035 | 0.908 | 0.159 | 0.587 | 0.218 | 0.511 | |
| P/D | 0.317 | 0.143 | 0.559 | 0.611 | **0.003** | -0.142 | **0.029** | 0.173 | 0.507 | 0.367 | 0.112 | |
| M/L | 0.061 | 0.759 | 0.398 | -0.333 | 0.712 | -0.352 | 0.233 | 0.047 | 0.877 | 0.204 | 0.550 | |
| **12% of gait cycle** | | | | | | | | | | | | |
| AB/AD | 0.119 | -0.806 | 0.397 | 1.079 | 0.270 | 0.196 | 0.522 | -0.176 | 0.544 | 0.384 | 0.233 |  |
| IR/ER | 0.022 | 0.196 | 0.863 | 0.459 | 0.692 | -0.164 | 0.596 | -0.393 | 0.184 | 0.143 | 0.682 |  |
| F/E | 0.007 | 0.379 | 0.806 | -0.348 | 0.818 | -0.255 | 0.409 | -0.089 | 0.775 | -0.683 | **0.032** |  |
| A/P | 0.090 | -0.263 | 0.882 | -1.708 | 0.351 | 0.112 | 0.708 | 0.086 | 0.773 | -0.292 | 0.379 |  |
| P/D | 0.103 | 1.099 | 0.576 | -2.251 | 0.270 | -0.035 | 0.908 | 0.159 | 0.587 | 0.218 | 0.511 |  |
| M/L | 0.078 | 0.209 | 0.840 | -1.037 | 0.336 | -0.040 | 0.894 | 0.169 | 0.570 | -0.378 | 0.252 |  |
| **52% of gait cycle** | | | | | | | | | | | | |
| AB/AD | 0.272 | -1.725 | 0.089 | 1.487 | 0.143 | 0.264 | 0.314 | 0.057 | 0.830 | 0.268 | 0.366 | |
| IR/ER | 0.165 | -0.203 | 0.818 | 1.330 | 0.156 | -0.128 | 0.655 | 0.155 | 0.584 | -0.514 | 0.090 | |
| F/E | 0.362 | -0.139 | 0.553 | -0.274 | 0.234 | -0.132 | 0.601 | -0.588 | **0.018** | -0.028 | 0.906 | |
| A/P | 0.180 | -2.667 | 0.181 | -0.524 | 0.789 | 0.077 | 0.786 | 0.014 | 0.959 | 0.225 | 0.478 | |
| P/D | 0.089 | 2.325 | 0.304 | -1.114 | 0.623 | -0.224 | 0.449 | 0.283 | 0.331 | 0.013 | 0.970 | |
| M/L | 0.246 | 2.598 | 0.071 | -0.851 | 0.536 | 0.063 | 0.817 | -0.121 | 0.653 | -0.086 | 0.779 | |
| **62% of gait cycle** | | | | | | | | | | | | |
| AB/AD | 0.102 | 0.790 | 0.588 | -1.362 | 0.262 | 0.297 | 0.307 | -0.080 | 0.786 | 0.184 | 0.581 | |
| IR/ER | 0.069 | -0.793 | 0.376 | 0.433 | 0.631 | -0.372 | 0.204 | -0.028 | 0.927 | -0.346 | 0.300 | |
| F/E | 0.005 | -0.370 | 0.892 | -0.370 | 0.894 | 0.109 | 0.727 | -0.048 | 0.877 | -0.264 | 0.450 | |
| A/P | 0.188 | -3.928 | 0.150 | -0.262 | 0.921 | 0.064 | 0.821 | 0.373 | 0.165 | 0.125 | 0.696 | |
| P/D | 0.378 | 0.197 | 0.394 | 0.198 | 0.388 | -0.166 | **0.015** | 0.185 | 0.456 | 0.184 | 0.423 | |
| M/L | 0.293 | 2.762 | 0.052 | -0.237 | 0.858 | 0.120 | 0.648 | -0.049 | 0.850 | -0.106 | 0.720 | |
| **75% of gait cycle** | | | | | | | | | | | | |
| AB/AD | 0.169 | 0.685 | 0.282 | 0.404 | 0.527 | 0.143 | 0.615 | -0.131 | 0.642 | 0.237 | 0.458 | |
| IR/ER | 0.303 | -0.259 | 0.284 | 0.127 | 0.608 | -0.039 | 0.884 | -0.321 | **0.034** | -0.201 | 0.412 | |
| F/E | 0.070 | 3.295 | 0.367 | -1.673 | 0.648 | 0.353 | 0.228 | 0.020 | 0.947 | 0.115 | 0.735 | |
| A/P | 0.106 | -0.819 | 0.649 | -1.577 | 0.394 | 0.063 | 0.831 | 0.163 | 0.578 | 0.212 | 0.523 | |
| P/D | 0.316 | 0.236 | 0.328 | -0.117 | 0.630 | -0.132 | **0.029** | 0.173 | 0.507 | 0.367 | 0.112 | |
| M/L | 0.098 | 1.090 | 0.489 | -1.675 | 0.302 | 0.049 | 0.870 | 0.358 | 0.210 | 0.175 | 0.600 | |
| **85% of gait cycle** | | | | | | | | | | | | |
| AB/AD | 0.350 | 2.214 | 0.083 | 1.125 | 0.344 | 0.101 | 0.691 | -0.178 | 0.473 | 0.382 | 0.162 | |
| IR/ER | 0.172 | 1.724 | 0.152 | -0.151 | 0.898 | -0.174 | 0.540 | 0.084 | 0.766 | -0.175 | 0.586 | |
| F/E | 0.024 | 1.773 | 0.603 | -0.382 | 0.912 | 0.410 | 0.168 | 0.055 | 0.859 | 0.109 | 0.756 | |
| A/P | 0.107 | -0.313 | 0.871 | -2.054 | 0.305 | 0.201 | 0.494 | 0.530 | 0.049 | 0.055 | 0.870 | |
| P/D | 0.142 | 0.883 | 0.550 | -2.044 | 0.187 | -0.389 | 0.163 | 0.012 | 0.967 | -0.231 | 0.477 | |
| M/L | 0.061 | -0.553 | 0.691 | -0.837 | 0.557 | -0.115 | 0.704 | 0.395 | 0.172 | -0.085 | 0.803 | |

Abbreviations: R², coefficient of determination; β (beta), regression coefficient. All angular values are presented in degrees (°). ROM, range of motion; AB/AD: adduction/abduction; IR/ER: internal/external; F/E: flexion/extension; A/P: anterior/posterior; P/D: proximal/distal; M/L: Medial/Lateral (the same below). Bold values indicate a statistically significant difference compared with healthy subjects (p < 0.05).
